# Circular RNA CDR1as Inhibits the Metastasis of Gastric Cancer through Targeting miR-876-5p/GNG7 Axis

**DOI:** 10.1155/2021/5583029

**Published:** 2021-06-16

**Authors:** Jiajia Jiang, Rong Li, Junyi Wang, Jie Hou, Hui Qian, Wenrong Xu

**Affiliations:** ^1^Aoyang Institute of Cancer, Affiliated Aoyang Hospital of Jiangsu University, Zhangjiagang, Jiangsu 215600, China; ^2^Zhenjiang Key Laboratory of High Technology Research on Exosomes Foundation and Transformation Application, Jiangsu Key Laboratory of Medical Science and Laboratory Medicine, School of Medicine, Jiangsu University, Zhenjiang Jiangsu 212013, China

## Abstract

Circular RNA CDR1as has been demonstrated to participate in various cancer progressions as miRNA sponges. The exact underlying mechanisms of CDR1as on gastric cancer (GC) metastasis remain unknown. Here, we found that CDR1as knockdown facilitated GC cell migration and invasion while its overexpression inhibited the migration and invasion abilities of GC cells *in vitro* and *in vivo*. Moreover, epithelial-mesenchymal transition- (EMT-) associated proteins and MMP2 and MMP9 were downregulated by CDR1as. Bioinformatics analysis combined with dual-luciferase reporter gene assays, western blot, RT-qPCR analysis, and functional rescue experiments demonstrated that CDR1as served as a miR-876-5p sponge and upregulated the target gene GNG7 expression to suppress GC metastasis. In summary, our findings indicate that CDR1as suppresses GC metastasis through the CDR1as/miR-876-5p/GNG7 axis.

## 1. Introduction

Gastric cancer (GC) is the third most common cause of cancer-related death worldwide [1]. Distant metastasis is one of the leading causes as GC cells are prone to invade into surrounding organs or distant tissues through the lymph nodes or blood [2]. However, the exact underlying mechanisms of GC metastasis are still far from clear. As the standard treatment for metastatic GC, chemotherapy and radiation therapy have shown to improve life quality and prolong survival time of patients, but the side effects including drug resistance and systemic cytotoxicity make the five-year survival rate of GC patients still very low [3]. Thus, it is urgent to uncover the molecular mechanisms of GC metastasis and explore new therapeutic targets for GC treatment.

Circular RNAs (circRNAs) are a new type of noncoding RNA with covalently closed loop. They have stable circular structure, high abundance, and tissue specificity and can serve as critical regulators in various human diseases. Through binding with miRNAs or other molecules, circRNAs could regulate gene expression at transcriptional, posttranscriptional, and translational levels, making them to be a novel potential therapeutic target [4]. CircRNA CDR1as, derived from the cerebellar degeneration-associated antigen 1 (CDR1) gene, has been identified as a miR-7 sponge in many diseases including Alzheimer's disease [5], myocardial infarction [6], osteoarthritis [7], and multiple types of cancer including liver cancer, non-small-cell lung cancer, and nasopharyngeal carcinoma [8–10]. Moreover, CDR1as participates in tumor metastasis with different mechanisms. For instance, CDR1as acts as a miR-135b-5p sponge and upregulates HIF1AN to suppress invasion and migration of ovarian cancer cells [11]. CDR1as epigenetic silencing mediated by IGF2BP3 promotes invasion and metastasis in melanoma [12]. However, the role and molecular mechanisms of CDR1as in GC metastasis remain to be uncovered.

In this study, we observed the suppressive effects of CDR1as on GC cell migration and invasion and identified miR-876-5p as a target miRNA of CDR1as to modulate GNG7 expression, which illustrates a new mechanism for CDR1as regulating GC metastasis and provides potential therapeutic targets for GC.

## 2. Materials and Methods

### 2.1. Cell Lines and Culture

Embryonic kidney cells 293T and human GC cell lines (SGC-7901, BGC-823, MKN-45, HGC-27, MGC-803, and AGS) were obtained from the Cell Bank of the Chinese Academy of Sciences (Shanghai, China). MKN-45, MGC-803, and 293T cells were cultured in high-glucose Dulbecco's modified Eagle's medium (DMEM; Gibco, Thermo Fisher Scientific, USA). HGC-27, BGC-823, and SGC-7901 cells were maintained in RPMI-1640 medium (Bioind, Israel). AGS cells were grown in DMEM/F-12 medium (Bioind, Israel). The above medium was supplemented with 10% fetal bovine serum (FBS; Bovogen, Australia). All cells were propagated humidified atmosphere with 5% CO_2_ at 37°C and tested for free of mycoplasma contamination periodically.

### 2.2. RNA Extraction and Real-Time Reverse Transcription Quantitative PCR (RT-qPCR)

Total RNA from cultured cells was extracted using TRIzol reagent (Invitrogen, Carlsbad, CA, USA) according to the manufacturers' protocol. For circRNA and mRNA expressions, RNA was reverse transcribed into cDNA by using HiScript 1st Strand cDNA Synthesis Kit (Vazyme, Nanjing, China) and then quantified by RT-qPCR with AceQ qPCR SYBR Green Master Mix (Vazyme, Nanjing, China). RT-qPCR reactions were run on ABI StepOnePlus Real-Time PCR Systems (Thermo Fisher Scientific, MA, USA) as follows: 95°C/5 min, 95°C/30 s, and 60°C/30 s for 40 cycles. Primer sequences were listed in Supplementary Table S1. RT-qPCR products were separated by 1.5% agarose gels and examined by UV irradiation. The relative expression level was calculated by 2^-△△Ct^ method with normalizing to the housekeeping *β*-actin [13].

### 2.3. Gene Silencing and Overexpression

Two different siRNAs targeting the CDR1as-specific junction sequence and two siRNAs targeting GNG7 were designed and synthesized (GenePharma, Shanghai, China) to perform gene silencing. The siRNA sequences were listed in Supplementary Table S2. The miRNAs (miR-203a, miR-876-5p, miR-3167, and miR-299-3p) mimics were obtained to overexpress miRNA level (GenePharma, Shanghai, China). GC cells were cultured at 37°C overnight. The siRNA or miRNA mimics (25 nM) were transfected into cells using Lipofectamine 2000 (Invitrogen, Carlsbad, CA, USA) in serum-free medium. After 6 h, the serum-free medium was changed with complete medium and cells were harvested for RNA analysis or functional experiments after 48 h and for protein analysis after 72 h. Lentivirus expression vector (Hanbio Biotechnology, Shanghai, China) was used to construct CDR1as stable overexpression cell lines including MKN-45, SGC-7901, and BGC-823. Cells were transduced with prepared lentivirus HBLV-CDR1as-GFP-PURO or HBLV-GFP-PURO (MOI = 30) and selected with 2 *μ*g/ml puromycin (Invitrogen, Carlsbad, CA, USA) at least two weeks. The intensity of GFP was evaluated by fluorescence microscope (BioTek, USA), and CDR1as level was determined by RT-qPCR analysis.

### 2.4. Cell Migration and Invasion Assays

Transfected GC cells (1 × 10^5^) in 200 *μ*l serum-free medium were, respectively, seeded into the upper chamber of the Transwell system (Corning, USA) without (migration assay) or with (invasion assay) Matrigel (BD Bioscience, USA) coating. Then, 600 *μ*l complete medium with 10% FBS was added to the lower chamber. After culturing for 12 h (migration assay) or 24 h (invasion assay), the cells that had migrated to the reverse side of the membrane were fixed in 4% paraformaldehyde, stained with 0.1% crystal violet, captured by microscopy, and counted. The nonmetastasis cells remaining on the upper chamber were cleared away.

### 2.5. Dual-Luciferase Reporter Gene Assay

The recombinant dual-luciferase reporter plasmid pmirGLO-CDR1as containing the full length of CDR1as cDNA was synthesized by Genscript (Nanjing, China). MGC-803 and 293T cells (2 × 10^4^/well) were seeded, respectively, overnight, and then, 400 ng luciferase reporter plasmids and 40 nM miRNA mimics were cotransfected into cells. Cells were collected and lysed after 48 h and then detected firefly and Renilla luciferase activity using Dual-Glo Luciferase Assay Kit (Promega) on GloMax 20/20 Luminometer (Promega).

### 2.6. Western Blot

Proteins from cells or tissues were extracted with RIPA lysis buffer (Pierce, MA, USA). Equal amount of protein was separated by SDS denatured polyacrylamide gel (SDS-PAGE) and transferred onto PVDF membrane (Millipore, Billerica, MA, USA). Then, the membrane was blocked with 5% (*w*/*v*) nonfat milk, incubated with specific primary antibodies at 4°C overnight and HRP-labelled secondary antibodies at 37°C for 1 h. The enhanced chemiluminescence system (ImageQuant LAS4000mini, GE, Japan) was used to visualize the protein band signals. The antibodies were as follows: rabbit anti-*β*-actin antibody (1 : 5000; Abclonal, Wuhan, China), rabbit anti-Vimentin antibody (1 : 800; Bioworld, USA), rabbit anti-E-cadherin antibody (1 : 300; Santa Cruz, USA), rabbit anti-N-Cadherin antibody (1 : 300; Cell Signaling, USA), rabbit anti-MMP2 antibody (1 : 300; Cell Signaling, USA), rabbit anti-MMP9 antibody (1 : 300; Cell Signaling, USA), rabbit anti-GNG7 antibody (1 : 500; ABclonal, USA), and Goat anti-Rabbit IgG Secondary Antibody (1 : 2000; Invitrogen, USA). The densitometry readings of each band were obtained using ImageJ program, and the fold change of indicated proteins was determined by normalizing to *β*-actin expression.

### 2.7. Metastasis Model in Mice

Male BALB/c nu/nu mice (Cavens, Changzhou, China) aged 4-6 weeks were bred under SPF conditions according to the institutional animal guidelines. An abdominal metastasis model was implemented as previously described [14]. CDR1as stably overexpressing MKN-45 cells (MKN-CDR1as) or its control cells (MKN-NC) (3 × 10^6^ cells in 200 *μ*l PBS per mouse) were injected into abdominal region of mice. Two weeks later, the mice were sacrificed and the metastasis nodules in abdominal region were counted and imaged. All animal experiments were approved by the Animal Use Ethics Committee of Jiangsu University.

### 2.8. Statistical Analysis

Data were analyzed by SPSS 21.0 (IBM, Chicago, IL, USA) and GraphPad Prism version 5.0 software (La Jolla, CA, USA). Student's *t*-test was used to assess the differences between two groups while one-way ANOVA was applied for differences among three or more experimental groups. The data of three independent experiments were presented as the mean ± SD, and *p* value < 0.05 was considered statistically significant.

## 3. Results

### 3.1. CDR1as Knockdown Promotes Migration and Invasion of GC Cells

**Figure 1:**
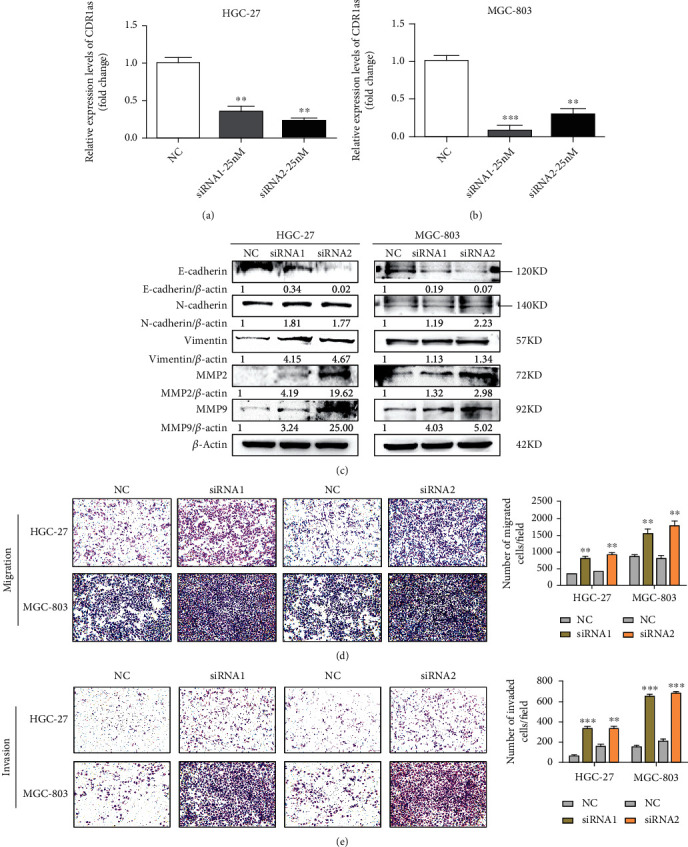
CDR1as knockdown promotes migration and invasion of GC cells. (a, b) RT-qPCR analysis of CDR1as level in HGC-27 and MGC-803 cells transfected with CDR1as and negative control (NC) siRNAs. (c) Western blot analysis for E-cadherin, N-cadherin, vimentin, and MMP expression levels after CDR1as silencing. (d, e) Migration and invasion assays for HGC-27 and MGC-803 cells after CDR1as knockdown. All values are expressed as the mean ± SD (*n* = 3) (^∗^*p* < 0.05, ^∗∗^*p* < 0.01, and ^∗∗∗^*p* < 0.001).

We firstly investigated the role of CDR1as on GC cell metastasis with loss of function strategies. Two separate siRNAs against CDR1as were transfected into HGC-27 and MGC-803 cells to knock down CDR1as level, and a nonspecific siRNA was used as control. RT-qPCR analysis confirmed the transfection efficiency (Figures 1(a) and 1(b)). Subsequently, the metastasis ability of GC cells was evaluated with Transwell assays. The results presented that CDR1as knockdown obviously facilitated the migration of HGC-27 and MGC-803 cells (Figure 1(d)) and effectively strengthened the invasion ability of GC cells (Figure 1(e)). To further explore the mechanism of metastasis in GC cells, epithelial-mesenchymal transition- (EMT-) associated proteins and matrix metalloproteinases (MMPs) were detected. The expression levels of mesenchymal markers N-cadherin and vimentin were increased while those of epithelial marker E-cadherin were decreased after CDR1as knockdown (Figure 1(c)). Meanwhile, knockdown of CDR1as promoted the MMP2 and MMP9 expressions (Figure 1(c)). These results indicate that CDR1as knockdown promotes the migration and invasion of GC cells probably through inducing the EMT process and MMP release.

### 3.2. CDR1as Overexpression Inhibits Metastasis of GC Cells In Vitro and In Vivo

**Figure 2:**
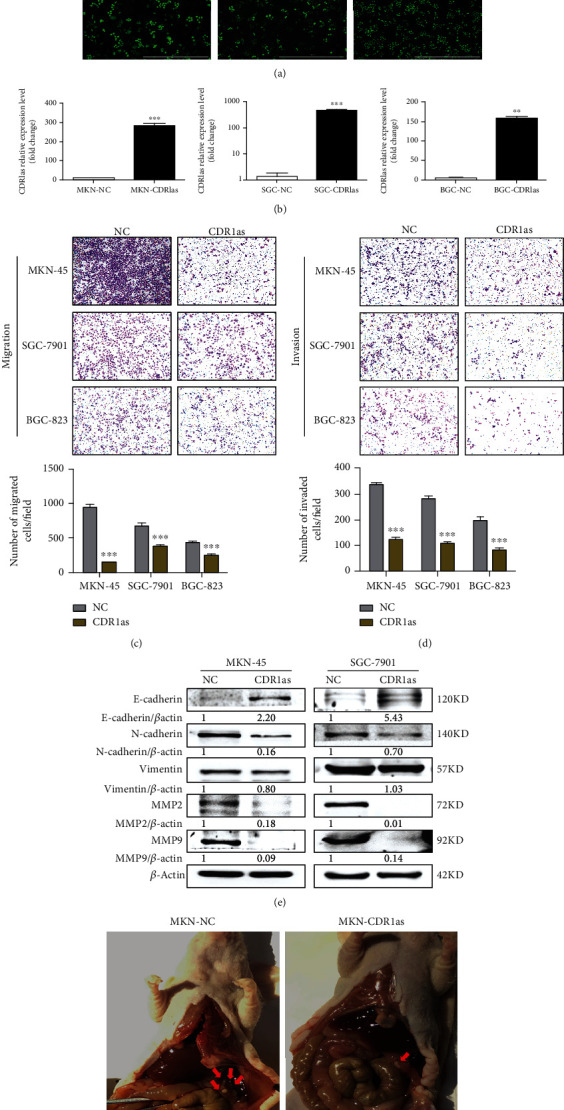
CDR1as overexpression inhibits metastasis of GC cells in vitro and in vivo. (a) The fluorescence intensity of GFP in MKN-45, SGC-7901, and BGC-823 cells after lentivirus-mediated transfection. (b) RT-qPCR analysis of CDR1as level in CDR1as stably overexpressing cell lines and control cells. (c, d) Migration and invasion assays in MKN-45, SGC-7901, and BGC-823 cells with CDR1as overexpression. (e) Western blot analysis for E-cadherin, N-cadherin, vimentin, and MMP expression levels after CDR1as overexpression. (f) The macroscopic appearance of the abdominal metastasis model in nude mice. Red arrows indicate abdominal metastatic nodules. All values are expressed as the mean ± SD (*n* = 3) (^∗^*p* < 0.05, ^∗∗^*p* < 0.01, and ^∗∗∗^*p* < 0.001).

To further confirm CDR1as effects on GC cell metastasis, we performed CDR1as overexpression in GC cell lines (MKN-45, SGC-7901, and BGC-823). GC cells were transduced with prepared lentivirus HBLV-CDR1as-GFP-PURO or HBLV-GFP-PURO to construct CDR1as stably overexpressing cells and corresponding control cells. The results indicated that most cells were transduced successfully and expressed GFP under the fluorescence microscope (Figure 2(a)). Also, CDR1as level in the experimental groups was over 100-fold higher than that in control groups by RT-qPCR (Figure 2(b)). Then, Transwell assays showed that the number of migrated and invaded GC cells with CDR1as overexpression was significantly lower than that of control cells (Figures 2(c) and 2(d)). Moreover, western blot analysis presented that the levels of N-cadherin, vimentin, MMP2, and MMP9 in CDR1as overexpressing GC cells were lower while E-cadherin were higher than that in control cells (Figure 2(e)), which was opposite to the effects observed with CDR1as knockdown. Additionally, in order to evaluate CDR1as effects on GC metastasis *in vivo*, the abdominal metastasis model in nude mice was constructed with CDR1as stably overexpressing MKN-45 cells (MKN-CDR1as) and its control cells (MKN-NC). We found that the number of abdominal metastatic nodules was reduced in mouse bearing MKN-CDR1as cells compared with MKN-NC cells (Figure 2(f)). Meanwhile, we also noticed that the volume of abdominal metastatic nodules in the MKN-CDR1as group was higher than that in the MKN-CDR1as group. These results suggest that CDR1as overexpression inhibits GC cell metastasis *in vitro* and *in vivo*.

### 3.3. miR-876-5p Reverses CDR1as to Promote Migration and Invasion of GC Cells

**Figure 3:**
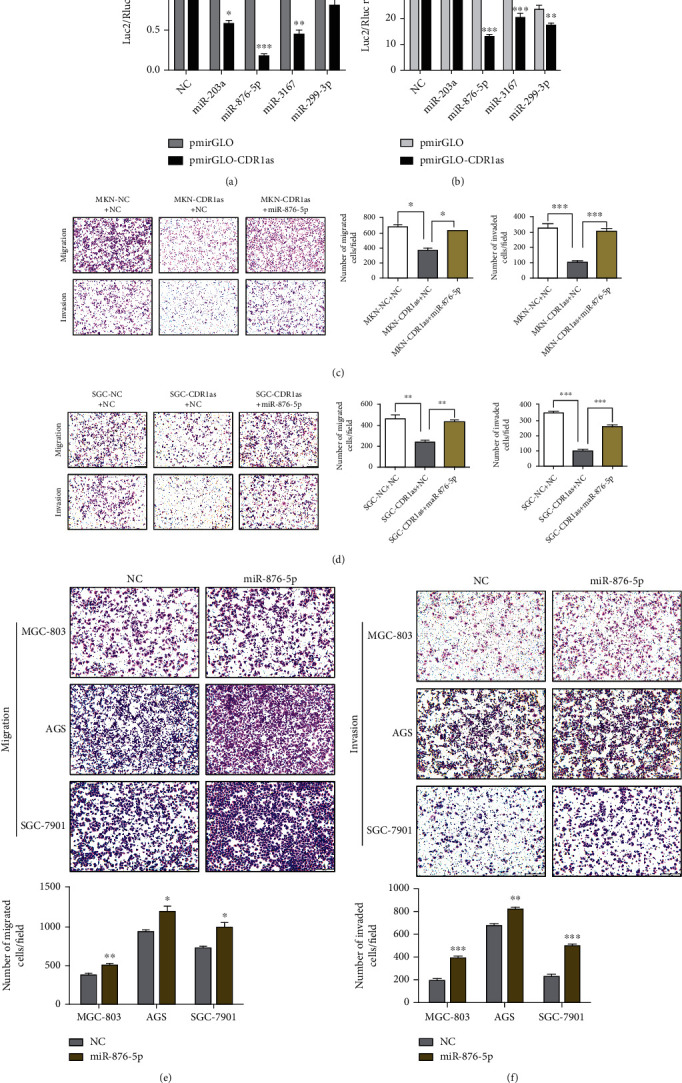
miR-876-5p reverses CDR1as to promote migration and invasion of GC cells. (a, b) Dual-luciferase reporter assays in MGC-803 and 293T cells to determine binding ability of potential miRNA and CDR1as. (c, d) Migration and invasion assays in MKN-45 and SGC-7901 cells with CDR1as overexpression cotransfected by miR-876-5p mimics. (e, f) Migration and invasion assays in MGC-803, AGS, and SGC-7901 cells transfected with miR-876-5p mimics. All values are expressed as the mean ± SD (*n* = 3) (^∗^*p* < 0.05, ^∗∗^*p* < 0.01, and ^∗∗∗^*p* < 0.001).

To identify the new miRNA targets of CDR1as apart from miR-7, we searched on starBase v2.0 (http://starbase.sysu.edu.cn/) and selected miR-203a, miR-876-5p, miR-3167, and miR-299-3p as candidates according to the value of biComplex and clipReadNum and the number of targetSites. Dual-luciferase reporter gene assays showed that overexpression of these miRNAs, especially miR-876-5p, significantly decreased the luciferase activity of reporter gene containing CDR1as sequence in MGC-803 and 293T cells, confirming the binding ability of CDR1as and miR-876-5p (Figures 3(a) and 3(b)). Furthermore, rescue experiments in MGC-803 and MKN-45 cells were performed. We found that the migration and invasion abilities of GC cells were inhibited by CDR1as while miR-876-5p observably reversed this effects (Figures 3(c) and 3(d)). Additionally, overexpression of miR-876-5p with miRNA mimics markedly strengthened the metastasis ability of GC cells, which is consistent with the effects induced by CDR1as silencing (Figures 3(e) and 3(f)). Altogether, these results infer that miR-876-5p may be a target of CDR1as for regulating migration and invasion of GC cells.

### 3.4. CDR1as Upregulates GNG7, a Target of miR-876-5p

**Figure 4:**
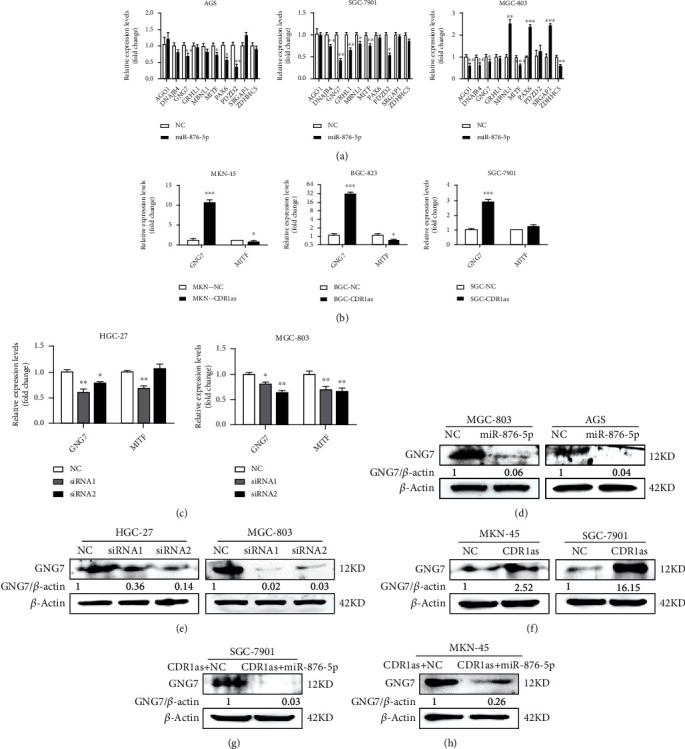
CDR1as upregulates GNG7, a target of miR-876-5p. (a) RT-qPCR analysis of ten potential target mRNA levels of miR-876-5p in three GC cells after transfection with miR-876-5p mimics. (b, c) RT-qPCR analysis of GNG7 and MITF expression levels in GC cells with CDR1as overexpression and knockdown. (d) Western blot analysis for GNG7 protein level in GC cells transfected with miR-876-5p mimics. (e, f) Western blot analysis for GNG7 protein level in GC cells with CDR1as knockdown and overexpression. (g, h) Western blot analysis for GNG7 protein level in CDR1as overexpressing GC cells cotransfected with miR-876-5p mimics. All values are expressed as the mean ± SD (*n* = 3) (^∗^*p* < 0.05, ^∗∗^*p* < 0.01, and ^∗∗∗^*p* < 0.001).

To determine the downstream target mRNA of miR-876-5p, starBase v2.0 was used to predict over 3,000 target mRNAs from 7 databases and 48 mRNAs were selected according to the coincidence degree (Supplementary Table S3). As miR-876-5p exerted promotive effects on GC cell metastasis, we mainly focused on tumor suppressive gene and thus selected 10 potential targets (Supplementary Table S3). After transfection with miR-876-5p mimics in AGS, SGC-7901, and MGC-803 cells, only GNG7 and MITF mRNA levels were reduced in all cell lines, suggesting that they might be target genes of miR-876-5p (Figure 4(a)). To identify which gene was the target of CDR1as when sponging miR-299-3p, we further detected their transcription levels after CDR1as overexpression and knockdown. GNG7 was the only gene whose expression was significantly increased in MKN-45, BGC-823, and SGC-7901 with CDR1as overexpression (Figure 4(b)). Meanwhile, CDR1as knockdown decreased GNG7 levels in HGC-27 and MGC-803 cells (Figure 4(c)). Western blot analysis also showed that miR-876 mimics suppressed the GNG7 protein level in GC cells (Figure 4(d)), and CDR1as knockdown downregulated while its overexpression upregulated GNG7 protein level (Figures 4(e) and 4(f)). Furthermore, upregulation of GNG7 protein level in CDR1as overexpressing GC cells was reversed by cotransfection with miR-876-5p (Figures 4(g) and 4(h)). Taken together, GNG7 is identified as a target gene of miR-876-5p and upregulated by CDR1as in GC cells.

### 3.5. Knockdown of GNG7 Reverses CDR1as to Promote Migration and Invasion of GC Cells

**Figure 5:**
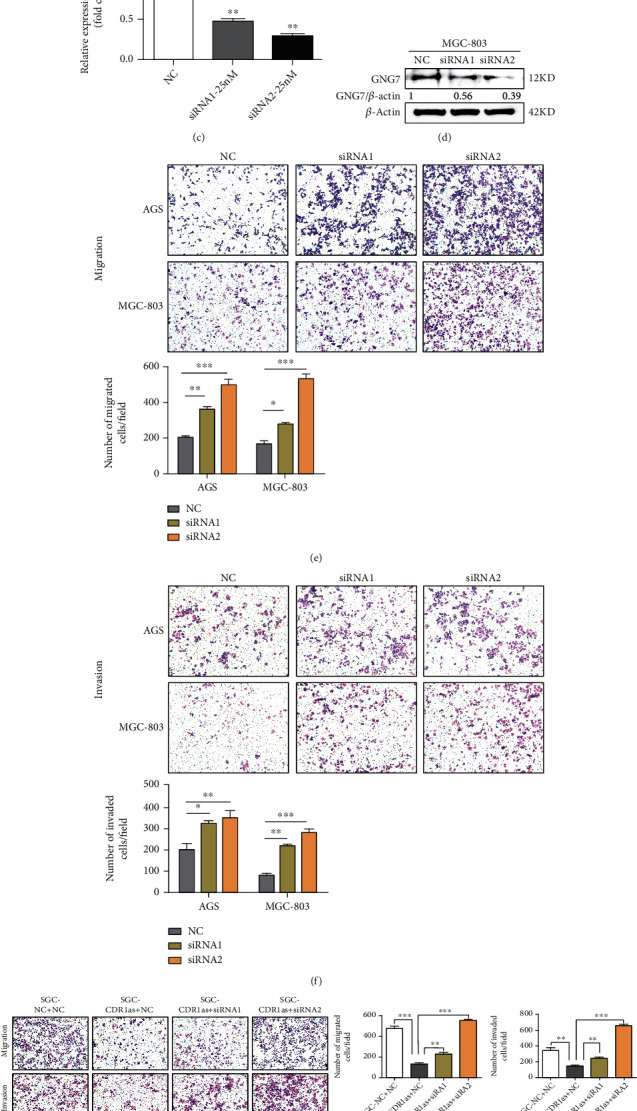
Knockdown of GNG7 reserves CDR1as to promote migration and invasion of GC cells. (a, c) RT-qPCR analysis of the GNG7 transcription level after transfection with GNG7 and negative control siRNAs. (b, d) Western blot analysis for the GNG7 protein level after transfection with GNG7 and negative control siRNAs. (e, f) Migration and invasion assays of GC cells after GNG7 knockdown. (g, h) Migration and invasion assays of CDR1as overexpressing GC cells cotransfected with GNG7 siRNAs. All values are expressed as the mean ± SD (*n* = 3) (^∗^*p* < 0.05, ^∗∗^*p* < 0.01, and ^∗∗∗^*p* < 0.001).

In order to identify whether GNG7 was an important regulator in CDR1as-mediated tumor suppressive effects, two different siRNAs were designed and transfected into AGS and MGC-803 cells to knockdown GNG7. RT-qPCR and western blot analysis confirmed the knockdown of GNG7 in GC cells (Figure 5(a)–5(d) ). After transfection, the migration and invasion abilities of GC cells were obviously promoted with GNG7 silencing (Figures 5(e) and 5(f)). Moreover, when we knockdown GNG7 in CDR1as overexpressing GC cells, the suppressive effects of CDR1as on cell migration and invasion were reduced or even reversed (Figures 5(g) and 5(h)). Therefore, these results suggest that GNG7 can mediate tumor suppressive effects of CDR1as on GC cell metastasis.

## 4. Discussion

Tumor metastasis is the pivotal determinant of poor outcomes for many cancer patients including GC [15]. It is a multistep cell biological process in which EMT and enhanced MMP function are important for the local invasion of primary tumor cells into surrounding tissues and far distance [2]. CDR1as, a hotspot in circRNA research field, has been discovered to exert different regulatory effects on tumor metastasis in different cancer types. It promotes cell metastasis in squamous cell carcinoma [16], non-small-cell lung cancer [17], and pancreatic cancer [18] while suppressing cell metastasis in ovarian cancer [11] and melanoma [12]. In the present study, CDR1as was observed to suppress GC cell metastasis with gain-of- and loss-of-function experiments. Meanwhile, EMT-associated proteins and MMPs were also downregulated. The abdominal metastasis model of nude mice was established to imitate the advanced metastatic GC, showing that CDR1as overexpression reduced the number of abdominal metastatic nodules. Although only one pair of nude mice was used, the suppressive effect of CDR1as on GC metastasis in nude mice was markedly. Thus, CDR1as suppresses GC cell metastasis probably through regulating MMP release and EMT process.

CDR1as has been demonstrated to regulate tumor progression as miRNA sponges to derepress downstream target mRNAs. Many miRNAs have been identified as the target of CDR1as especially miR-7. For example, CDR1as accelerates colorectal cancer progression through sponging miR-7 to upregulate its target EGFR-RAF1 [19]. CDR1as acts as miR-7 sponges to upregulate targets of miR-7 including EGFR, CCNE1, and PIK3CD in non-small-cell lung cancer [9]. Furthermore, miR-1270 is validated as a target of CDR1as in accelerating hepatocellular carcinoma [20]. Moreover, CDR1as suppresses ovarian cancer progression as miR-135b-5p sponges to upregulate HIF1AN expression [11]. In this study, miR-876-5p was identified as a target of CDR1as. Bioinformatics prediction combined with dual-luciferase reporter gene assays confirmed the binding ability of CDR1as and miR-876-5p. Transwell assays showed the promotive effects of miR-876-5p on GC cell migration and invasion, which was opposite to the effects of CDR1as. When CDR1as overexpressing cells were rescued with miR-876-5p, the suppressive effects of CDR1as on cell metastasis were obviously reversed. Therefore, CDR1as suppresses GC cell metastasis through acting as a miR-876-5p sponge.

G protein *γ* subunit 7 (GNG7), a member of large G *γ* family, has been reported to be downregulated in various cancers including pancreatic cancer [21], oesophageal cancer [22], and gastrointestinal tract cancer [23]. The decrease of GNG7 expression is related to hypermethylation of the GNG7 promoter region [22, 24] or decreased enhancer of zeste homolog 2 (EZH2) and increased disabled homolog 2-interacting protein (DAB2IP) [25]. Moreover, most of studies indicate that GNG7 is a potential tumor suppressor. In oesophageal cancer, tumors with high GNG7 expression are less invasive than those with low GNG7 expression *in vitro* and *in vivo* [22]. In clear cell renal cell carcinoma, cells with lower GNG7 level exhibit an increase in G2/M cell cycle phase [25]. In gastrointestinal tract cancer, GNG7 suppresses cell growth *in vitro* and *in vivo* through upregulating the expression a cyclin-dependent kinase inhibitor p27^Kip1^ [24]. GNG7 can induce autophagy and cell death via suppressing MTOR pathway and inhibit cell division by regulating actin cytoskeleton [26]. In this study, GNG7 was identified as the target of miR-876-5p through bioinformatics prediction combined with RT-qPCR and western blot analysis. Meanwhile, CDR1as positively regulated the protein and mRNA levels of GNG7. Upregulated GNG7 levels in CDR1as overexpressing GC cells could be downregulated by rescued with miR-876-5p, suggesting CDR1as might inhibit miR-876-5p effects to upregulate GNG7 levels. Moreover, GNG7 might be a suppressive gene in regulating GC metastasis as its knockdown promoted GC cell migration and invasion. When the expression of GNG7 in CDR1as overexpressing GC cells were knocked down, the suppressive effects on cell metastasis was reduced or even reversed, revealing that GNG7 is important for CDR1as to exert suppressive effects on GC metastasis.

Molecularly targeted therapy including small molecular drugs or monoclonal antibodies targeting EGFR, VEGF, and HER2 has been developed due to the limitations of traditional cancer treatment [27]. However, tumor cells can become resistant and some drugs are hard to develop due to the target's structure [28]. As natural miRNA sponges to regulate tumor-related proteins, circRNAs have great potential to be developed as effective therapeutic targets. Manipulating circRNA levels to suppress tumor progression might be a useful method. In our study, lentiviral vector was applied to increase CDR1as level in GC cells and significantly suppressed GC metastasis *in vitro* and *in vivo*. Additionally, a kind of synthetic circRNA containing miR-21 binding sites has been proved to suppress GC cell growth in vitro [29], suggesting that synthetic CDR1as sponges might be a simple, effective, and convenient therapeutic strategy. The safety and efficacy of these proposed strategies remain to be further validated.

In conclusion, CDR1as plays an important suppressive role in GC metastasis through sponging miR-876-5p to upregulate the GNG7 expression. These findings elucidate a novel mechanism for GC metastasis and provide a potential therapeutic strategy for GC treatment in future.

## Data Availability

The data used to support the findings of this study are available from the corresponding author request.

## Supplementary Materials

Supplementary Materials Supplementary Table S1 and S2: the primer and oligonucleotide sequences. Supplementary Table S3: the prediction for target mRNAs of miR-876-5p using starBase v2.0.
