# Supplementary material for: Circular RNA CDR1as Inhibits the Metastasis of Gastric Cancer through Targeting miR-876-5p/GNG7 Axis
Source: Gastroenterol Res Pract. 2021 Jun 16;2021:5583029. doi: 10.1155/2021/5583029 (PMC8225434; doi:10.1155/2021/5583029)
Supplement: Supplementary Materials — Supplementary Table S1 and S2: the primer and oligonucleotide sequences. Supplementary Table S3: the prediction for target mRNAs of miR-876-5p using starBase v2.0. [file 5583029.f1.docx]

**Table S1. The sequences of gene-specific primers for RT-qPCR**

| Gene | Forward primer | Reverse primer |
| --- | --- | --- |
| CDR1as | ACGTCTCCAGTGTGCTGA | CTTGACACAGGTGCCATC |
| β-actin | CTCAGGAGGAGCAATGATCT | GACCTGTACGCCAACACAGT |
| AGO1 | TCGCCCTGCTAGCCATCAGACATT | TACAGCGCTGCCCAGCCACAA |
| DNAJB4 | CCAGCAGACATTGTTTTTATCATT | CCATCCAGTGTTGGTACATTAATT |
| GNG7 | CGTCTGACCTCATGAGCTACTGTGA | CAAGGTTTCTTGTCCTTAAAGGGGTTC |
| GRHL1 | CAAACGGCCAGTGTTGGTTC | TGCTCATCATCGCTTTGGTCG |
| MBNL1 | CTGCCGAACATCTGACTAGC | GCAACAGTGTGCAGTGGATT |
| MITF | CAGGCATGAACACACATTCAC | TCCATCAAGCCCAAGATTTC |
| PAX6 | CTGGAGAAAGAGTTTGAGAGGAC | GCTGTGGAATTGGCTGGTAG |
| PDZD2 | TCTGTACTGTGTACCTCACCAA | CCCTGCGCTTTTCACCATAG |
| SRGAP1 | GCCACCAATGCCTCAGTTTT | AAGGTTGTACTCCGCAGACA |
| ZDHHC5 | ACACCTCGGCTTGGCTACTA | GTTGGCTCCTTCAAGCTGTC |

**Table S2. The sequences of siRNAs for gene knockdown**

| siRNA | sense | antisense |
| --- | --- | --- |
| NC | UUCUCCGAACGUGUCACGUTT | ACGUGACACGUUCGGAGAATT |
| CDR1as siRNA1 | UCUGCAAUAUCCAGGGUUUTT | AAACCCUGGAUAUUGCAGATT |
| CDR1as siRNA2 | UAUCCAGGGUUUCCGAUGGTT | CCAUCGGAAACCCUGGAUATT |
| GNG7 siRNA1 | GCCACUAACAACAUAGCCCTT | GGGCUAUGUUGUUAGUGGCTT |
| GNG7 siRNA2 | GGUGGAACAGCUACGCAUATT | UAUGCGUAGCUGUUCCACCTT |

**Table S3. Potential target mRNAs of miR-876-5p predicted via starBase v2.0**

| miRNAname | geneID | geneName | PITA | RNA22 | miRmap | microT | miRanda | PicTar | TargetScan | **coincidence number** |
| --- | --- | --- | --- | --- | --- | --- | --- | --- | --- | --- |
| hsa-miR-876-5p | ENSG00000095787 | WAC | 1 | 0 | 1 | 1 | 1 | 1 | 1 | 6 |
| hsa-miR-876-5p | ENSG00000007372 | PAX6 | 1 | 0 | 1 | 1 | 1 | 1 | 1 | 6 |
| hsa-miR-876-5p | ENSG00000110429 | FBXO3 | 1 | 0 | 1 | 1 | 1 | 1 | 1 | 6 |
| hsa-miR-876-5p | ENSG00000156642 | NPTN | 1 | 0 | 1 | 1 | 1 | 1 | 1 | 6 |
| hsa-miR-876-5p | ENSG00000129993 | CBFA2T3 | 1 | 0 | 1 | 1 | 1 | 1 | 1 | 6 |
| hsa-miR-876-5p | ENSG00000213639 | PPP1CB | 1 | 0 | 1 | 1 | 1 | 1 | 1 | 6 |
| hsa-miR-876-5p | ENSG00000091436 | MAP3K20 | 1 | 0 | 1 | 1 | 1 | 1 | 1 | 6 |
| hsa-miR-876-5p | ENSG00000152601 | MBNL1 | 1 | 0 | 1 | 1 | 1 | 1 | 1 | 6 |
| hsa-miR-876-5p | ENSG00000113758 | DBN1 | 1 | 0 | 1 | 1 | 1 | 1 | 1 | 6 |
| hsa-miR-876-5p | ENSG00000163479 | SSR2 | 1 | 0 | 1 | 1 | 1 | 0 | 1 | 5 |
| hsa-miR-876-5p | ENSG00000163479 | SSR2 | 1 | 0 | 1 | 1 | 1 | 0 | 1 | 5 |
| hsa-miR-876-5p | ENSG00000153187 | HNRNPU | 1 | 0 | 1 | 1 | 0 | 1 | 1 | 5 |
| hsa-miR-876-5p | ENSG00000025800 | KPNA6 | 1 | 0 | 1 | 1 | 0 | 1 | 1 | 5 |
| hsa-miR-876-5p | ENSG00000092847 | AGO1 | 1 | 0 | 1 | 1 | 0 | 1 | 1 | 5 |
| hsa-miR-876-5p | ENSG00000092847 | AGO1 | 1 | 0 | 1 | 1 | 0 | 1 | 1 | 5 |
| hsa-miR-876-5p | ENSG00000092847 | AGO1 | 1 | 0 | 1 | 1 | 1 | 0 | 1 | 5 |
| hsa-miR-876-5p | ENSG00000162616 | DNAJB4 | 1 | 0 | 1 | 1 | 1 | 0 | 1 | 5 |
| hsa-miR-876-5p | ENSG00000086475 | SEPHS1 | 1 | 0 | 0 | 1 | 1 | 1 | 1 | 5 |
| hsa-miR-876-5p | ENSG00000227345 | PARG | 1 | 0 | 1 | 1 | 1 | 0 | 1 | 5 |
| hsa-miR-876-5p | ENSG00000099246 | RAB18 | 1 | 0 | 1 | 1 | 1 | 0 | 1 | 5 |
| hsa-miR-876-5p | ENSG00000156599 | ZDHHC5 | 1 | 0 | 1 | 1 | 1 | 0 | 1 | 5 |
| hsa-miR-876-5p | ENSG00000166881 | NEMP1 | 1 | 1 | 0 | 1 | 1 | 0 | 1 | 5 |
| hsa-miR-876-5p | ENSG00000187109 | NAP1L1 | 1 | 0 | 1 | 0 | 1 | 1 | 1 | 5 |
| hsa-miR-876-5p | ENSG00000157837 | SPPL3 | 1 | 0 | 1 | 1 | 1 | 0 | 1 | 5 |
| hsa-miR-876-5p | ENSG00000196935 | SRGAP1 | 1 | 0 | 1 | 1 | 0 | 1 | 1 | 5 |
| hsa-miR-876-5p | ENSG00000136111 | TBC1D4 | 1 | 0 | 1 | 1 | 1 | 0 | 1 | 5 |
| hsa-miR-876-5p | ENSG00000180008 | SOCS4 | 1 | 0 | 1 | 1 | 1 | 0 | 1 | 5 |
| hsa-miR-876-5p | ENSG00000129993 | CBFA2T3 | 1 | 0 | 1 | 1 | 1 | 0 | 1 | 5 |
| hsa-miR-876-5p | ENSG00000167182 | SP2 | 1 | 0 | 0 | 1 | 1 | 1 | 1 | 5 |
| hsa-miR-876-5p | ENSG00000154920 | EME1 | 1 | 0 | 1 | 1 | 1 | 0 | 1 | 5 |
| hsa-miR-876-5p | ENSG00000119547 | ONECUT2 | 1 | 0 | 1 | 1 | 0 | 1 | 1 | 5 |
| hsa-miR-876-5p | ENSG00000176533 | GNG7 | 1 | 1 | 1 | 1 | 1 | 0 | 0 | 5 |
| hsa-miR-876-5p | ENSG00000115137 | DNAJC27 | 1 | 0 | 1 | 1 | 1 | 0 | 1 | 5 |
| hsa-miR-876-5p | ENSG00000134317 | GRHL1 | 1 | 0 | 1 | 1 | 1 | 0 | 1 | 5 |
| hsa-miR-876-5p | ENSG00000132670 | PTPRA | 1 | 0 | 1 | 1 | 1 | 0 | 1 | 5 |
| hsa-miR-876-5p | ENSG00000114251 | WNT5A | 1 | 1 | 1 | 1 | 1 | 0 | 0 | 5 |
| hsa-miR-876-5p | ENSG00000187098 | MITF | 1 | 1 | 1 | 1 | 1 | 0 | 0 | 5 |
| hsa-miR-876-5p | ENSG00000008952 | SEC62 | 1 | 0 | 0 | 1 | 1 | 1 | 1 | 5 |
| hsa-miR-876-5p | ENSG00000164144 | ARFIP1 | 1 | 0 | 1 | 1 | 1 | 1 | 0 | 5 |
| hsa-miR-876-5p | ENSG00000129116 | PALLD | 1 | 1 | 1 | 1 | 1 | 0 | 0 | 5 |
| hsa-miR-876-5p | ENSG00000154122 | ANKH | 1 | 0 | 0 | 1 | 1 | 1 | 1 | 5 |
| hsa-miR-876-5p | ENSG00000081189 | MEF2C | 1 | 0 | 1 | 1 | 1 | 1 | 0 | 5 |
| hsa-miR-876-5p | ENSG00000133401 | PDZD2 | 1 | 0 | 0 | 1 | 1 | 1 | 1 | 5 |
| hsa-miR-876-5p | ENSG00000186687 | LYRM7 | 1 | 0 | 1 | 1 | 1 | 0 | 1 | 5 |
| hsa-miR-876-5p | ENSG00000181315 | ZNF322 | 1 | 0 | 1 | 1 | 1 | 0 | 1 | 5 |
| hsa-miR-876-5p | ENSG00000112218 | GPR63 | 1 | 0 | 1 | 1 | 1 | 0 | 1 | 5 |
| hsa-miR-876-5p | ENSG00000008083 | JARID2 | 1 | 0 | 0 | 1 | 1 | 1 | 1 | 5 |
| hsa-miR-876-5p | ENSG00000153814 | JAZF1 | 1 | 0 | 1 | 1 | 1 | 1 | 0 | 5 |
| hsa-miR-876-5p | ENSG00000197892 | KIF13B | 1 | 0 | 1 | 1 | 0 | 1 | 1 | 5 |
| hsa-miR-876-5p | ENSG00000175893 | ZDHHC21 | 1 | 0 | 0 | 1 | 1 | 1 | 1 | 5 |
| hsa-miR-876-5p | ENSG00000185963 | BICD2 | 1 | 0 | 0 | 1 | 1 | 1 | 1 | 5 |
| hsa-miR-876-5p | ENSG00000106799 | TGFBR1 | 1 | 0 | 0 | 1 | 1 | 1 | 1 | 5 |
